# Fast formation of a supramolecular ion gel/solvoplastic elastomer with excellent stretchability

**DOI:** 10.1098/rsos.180271

**Published:** 2018-06-20

**Authors:** Shishun Bai, Xin Wang, Jaana Vapaavuori, Xianru He

**Affiliations:** 1 School of Materials Science and Engineering, Energy Polymer Research Center, Southwest Petroleum University, 8 Xindu Avenue, Chengdu, Sichuan 610500, China; 2 Département de chimie, Université de Montréal, C.P. 6128, succursale Centre-Ville, Montréal, Quebec H3C 3J7, Canada

**Keywords:** supramolecular ion gel, random copolymer, quaternization, solvoplastic elastomer

## Abstract

This study describes a simple yet efficient approach for the preparation of an ionic gel that is also elastomeric in its solid-state bulk form. A series of poly(2-(diethylamino)ethyl methacrylate-*co*-lauryl methacrylate) P(DMAEMA-*co*-LMA) copolymers were synthesized first by radical polymerization. Quaternization of the PDMAEMA component in tetrahydrofuran enables the formation of supramolecular network, giving rise to an ion gel. An elastomer with an elongation at break of over 600% was obtained from the gel. The elastomer, connected by supramolecular ionic cross-links, is solvoplastic in certain solvents. The simple yet efficient approach of the formation of ion-gel and the dried elastomer allows fast preparation of both gel-like and solid-state elastic materials for various applications where recyclability is required.

## 1. Introduction

Gels are highly useful materials possessing properties of both liquids and solid materials. Ion gels, commonly formed from polymers in an ionic liquid, were found to have unique properties as polymer electrolytes, particularly when used as transistors in electronics due to their high ion conductivity [1–3]. Supramolecular ion-gels have drawn specific research attention over the years mostly because of their facile preparation and tunable functionality [4,5]. Supramolecularly cross-linked gels have shown potential for self-healing [6], drug release [7] and external stimuli responsiveness [8–12]. The introduction of physically cross-linked networks through quaternization is a very convenient and effective method [13–15]. The combination of supramolecular strategy and ion conductivity allows the tuning of the physical shape of the gels/elastomers and is of interest for wearable electronics [16].

Most of the ion-gels reported to date have been prepared by dissolving a triblock copolymer in an ionic liquid [17–19]. The preparation of a block copolymer, however, is more labour-intensive and less cost-effective than statistical/random copolymerization. It is worth mentioning that random copolymer has more dispersed monomer combination and more convenient preparation process, which can self-assemble to different morphological aggregates, such as spherical micelles and vesicles, in a selective solvent [20–22]. On the other hand, use of ionic liquid also complicates the procedure, for that ionic liquids need specific synthesis and care, which sometimes can also bring certain environmental issues [10,23,24]. In addition, most of the ion-gels were primarily used as transistors by taking advantage of their ionic conductivity. In cases where mechanical properties are required, for example as free standing films, ion-gels that can withstand external forces are required. In order to obtain an ionized polymer, quaternization of contralateral chains is convenient and efficient [25,26].

In general, there is little research on the mechanical properties of the solid-state materials that are formed upon collapse of the gel network through solvent removal. Thus, in order to study the mechanical properties of gel products after solvent evaporation, we designed and synthesized a series of copolymers of lauryl methacrylate (LMA) and quaternized 2-(diethylamino)ethyl methacrylate (DMAEMA) that gave mechanically robust free-standing films ideal for investigations of mechanical properties in solid state. The mechanical properties of the gel were examined by rheometry. The bulk form of the gel, which is an elastomer, was also investigated by tensile test. The elastomer could also be dissolved and recast into free standing film with mechanical properties that are similar to those of the initial film.

## 2. Experimental

### 2.1. Materials

All materials were obtained from Chengdu Kelong Chemical Inc. unless otherwise indicated. Anisole, iodomethane, *n*-hexane, DMAEMA and LMA were obtained from the Aladdin Corporation and passed through an aluminium oxide column just before polymerization to eliminate the inhibitor. 2,2′-Azobis(2-methylpropionitrile) (AIBN) was recrystallized once from ethanol before use.

### 2.2. Synthesis of P(DMAEMA-*co*-LMA) copolymers

P(DMAEMA-*co*-LMA), abbreviated P_*x*_ (P for starting polymers), where *x* is a number distinguishing the four polymers, in order of their PDMAEMA content. The preparation of P1 is given as an example. In a 50 ml Schlenk flask, DMAEMA (5.0 g, 31.8 mmol), LMA (5.0 g, 19.6 mmol) and AIBN (6.4 mg, 0.1 mmol) were mixed under stirring for 10 min. The flask was then put in a heating bath at 65°C for 5 h. After concentrating the solution, the product was precipitated once in *n*-hexane cooled by refrigerator. The sample was dried in a vacuum oven at 50°C for 2 days and kept in sealed vial. Monomer feed ratios of the copolymers are summarized in table 1. ^1^H NMR (400 MHz, CDCl_3_), *δ* (TMS, ppm): 4.09 (d, *J* = 3.81 Hz, 2H, O-CH_2_), 3.94 (d, *J* = 5.61 Hz, 2H, O-CH_2_), 2.59 (d, *J* = 5.52 Hz, 2H, CH_2_-N), 2.31 (d, *J* = 5.92 Hz, 6H, N-(CH_3_)_2_), 1.82 (s, 2H, CH_2_), 1.63 (s, 2H, CH_2_), 1.45 (s, 2H, CH_2_-CH_3_), 1.29 (s, 2H, CH_2_), 1.04 (s, 3H, CH_3_-CH_2_), 0.91 (t, 3H, *J* = 6.56 Hz, CH_3_).

**Table 1.** Monomer feed ratio of P(DMAEMA-*co*-LMA)s and their quaternized copolymers.

starting copolymer	LMA/DMAEMA wt.	monomer/anisole vol.	corresponding quaternized copolymer
P1	1	1	Q1
P2	3	1	Q2
P3	5	1	Q3
P4	10	1	Q4

### 2.3. Quaternization of P(DMAEMA-*co*-LMA) copolymers

P(DMAEMAQ-*co*-LMA), abbreviated Q_*x*_ (Q for quaternized polymers), where *x* is the same number as in its corresponding starting polymer P_*x*_. For the study of the elasticity, the gel of Q4 was dried to totally remove the solvent and defined as Q4s. The random copolymers were quaternized according to a literature procedure [27]. The preparation of Q1 is given as an example. A solution of P1 (0.58 g) in tetrahydrofuran (THF; 5 ml) was stirred for 10 min in a 25 ml Schlenk flask. Then iodomethane (3 eq, 34.44 µl) was quickly added under stirring. Gelation occurred at about 3 min after completion of addition. ^1^H NMR (400 MHz, DMF-*d*_7_), *δ* (TMS, ppm): 4.64 (2H, s, O-CH_2_), 4.01 (s, 2H, O-CH_2_), 4.26 (s, 2H, CH_2_-N^+^), 3.56 (d, 9H, *J* = 6.57 Hz, N^+^-(CH_3_)_3_), 1.99 (br, 2H, CH_2_), 1.68 (s, 2H, CH_2_), 1.40 (s, 2H, CH_2_-CH_3_), 1.32 (s, 2H, CH_2_), 1.05 (br, 3H, CH_3_-CH_2_), 0.90 (s, 3H, CH_3_).

**Scheme 1.**
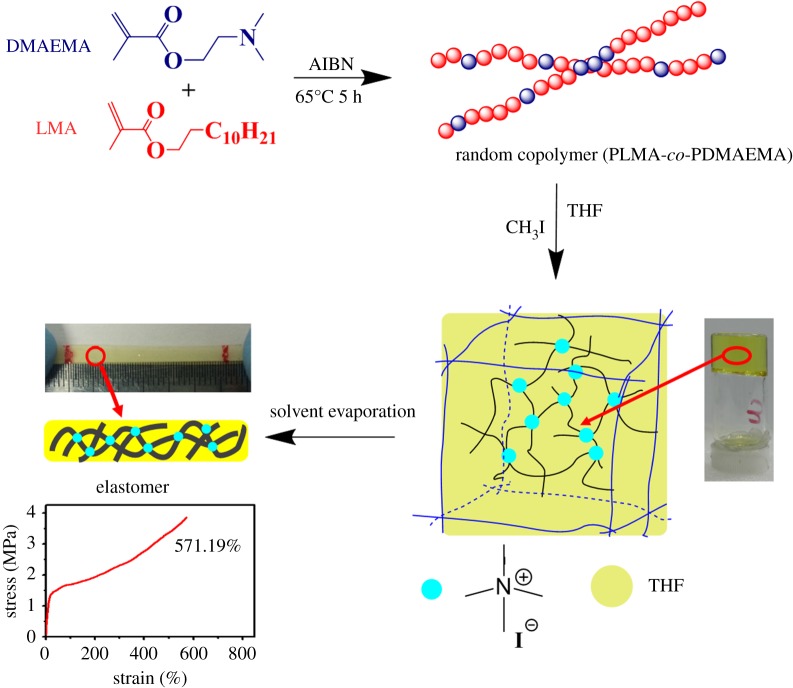
Schematic illustration of the preparation of starting copolymer and the subsequent gelation leading to the supramolecular ion-gel (elastomer when in bulk form).

The synthetic routes of starting copolymers and their corresponding quaternized copolymer ion-gels are given in scheme 1.

### 2.4. Instrumentation

Fourier transform infrared spectra on KBr pellets were recorded with a WQF-600 spectrometer, at a resolution of 4 cm^−1^. Each sample was scanned 32 times. For Q1, the powder was thoroughly dried and ground with KBr, followed by pressing into round pellet. All the other samples, i.e. P1–P4 and Q2–Q4, were first dissolved in THF and then coated on dried KBr pellet prepared in advance. Proton nuclear magnetic resonance (^1^H NMR) spectra in deuterated chloroform (CDCl_3_) were obtained with a Bruker AVANCE III 400 MHz spectrometer. Number average molecular weight (*M*_n_), weight average molecular weights (*M*_w_) and molecular-weight dispersities were measured by gel permeation chromatography analysis with an Agilent 1100 series liquid chromatograph (Agilent Technologies, USA) equipped with a refractive index detector (G 1362 A) and a series of 10 000 and 100 Å pore sized polydivinylbenzene columns thermostatically controlled at 35°C. THF with 3 vol% triethylamine was used as the eluent. The flow rate was 1 ml min^−1^, and the solution concentration was about 3 mg ml^−1^. The column was calibrated with polystyrene standards (*M*_w_). Thermogravimetric analysis (TGA) was performed using a TA Instruments SDTA851^e^ analyser under a nitrogen atmosphere from room temperature to 500°C at a heating rate of 10°C min^−1^. Differential scanning calorimetry (DSC) was conducted with a TA Q20 calorimeter. 6–10 mg of sample was first heated to 40°C from room temperature at 10°C min^−1^ and stabilized for 5 min, followed by cooling to −70°C at 10°C min^−1^. The heating–cooling cycle was repeated for another two times. The heat flow of the heating process was recorded. The glass transition temperature (*T*_g_) was determined as the midpoint of the glass transition region of the heat flow curve. Rheological studies were performed on freshly prepared copolymer ion-gels using a TA AR 2000ex rotational rheometer. Silicon oil was used during measurements to cover the samples and limit THF evaporation. Mechanical measurement was done with a YG 001 A Jigao stretching instrument. The sample with a dimension of 26 × 3 × 0.26 mm (L × W × T) was stretched to 50%, 100%, 150%, 200%, 250%, 300%, 350% and 400% at 20 mm min^−1^, where it was kept for 5 min, followed by removal of external force to allow the recovery to its original state. Lost recovery was then calculated based on the length before and after stretching.

## 3. Results and discussion

### 3.1. Synthesis and characterization

To obtain starting copolymers with suitable softness and subsequent elasticity, as well as to facilitate the synthesis, LMA, whose homopolymer has a reported *T*_g_ of about −70°C [28], was chosen as the soft segment, whereas DMAEMA was used as the segments to form cross-links after ionic modification (quaternization). The component of the starting copolymers was regulated by varying the monomer feed ratio to give rise to copolymers with different softness. The composition of the copolymers was determined by ^1^H NMR spectra as shown in electronic supplementary material, figures S1 and S2.

**Figure 1.**
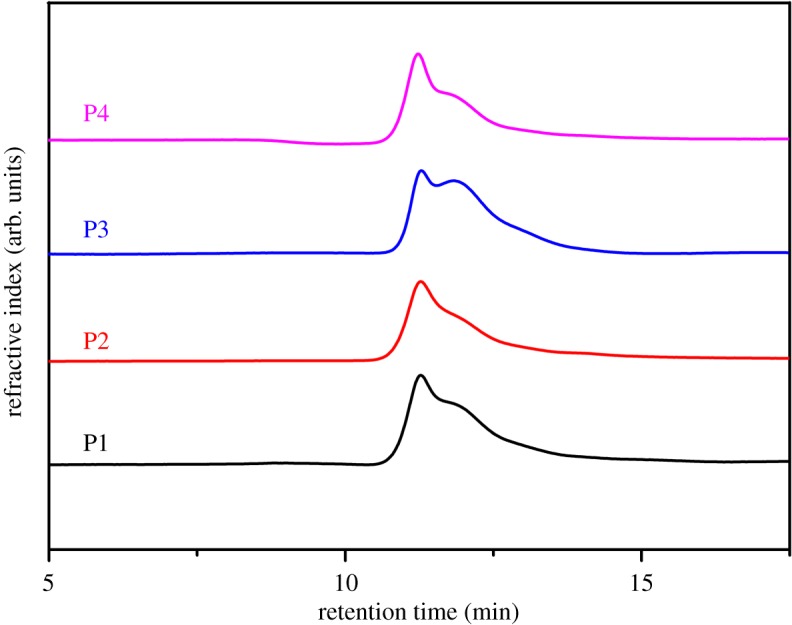
Gel permeation chromatography elugrams of P1, P2, P3 and P4.

The exact component weight ratio of each copolymer was calculated from equation (3.1):

3.1 $$\frac{M_{\mathrm{LMA}}}{M_{\mathrm{DMAEMA}}}=\frac{(S_{c+h}-S_{d})\times 254}{S_{d}\times 157},$$

where *S_c+h_* is the total integral area of peaks *c* and *h*, and *S_d_* is the integral area of peak *d*, as shown in electronic supplementary material, figure S1. The molecular weight of DMAEMA and LMA is taken as 254 and 157 g mol^−1^, respectively. The results from ^1^H NMR agree well with the theoretical values within 3% of experimental error as shown in electronic supplementary material, table S1, suggesting a successful preparation of the copolymers with desired component ratio. Furthermore, all the weight-average molecular weights and polydispersities of the starting copolymers (P1–P4) are also similar to each other, as shown in figure 1. All the polymers have a relatively wide molecular weight distribution, because they are prepared by traditional free radical polymerization. Larger molecular weight distribution of polymer is generally observed in uncontrolled traditional free radical polymerization, as compared to living free radical polymerization where the polymer chains are equally growing. In this study, the simplicity of preparation of gels and elastomers is critical and therefore the more facile traditional radical polymerization was employed. The degree of quaternization of every starting copolymers was determined by ^1^H NMR (electronic supplementary material, figures S3 and S4). The calculated results are shown in electronic supplementary material, table S2.

**Figure 2.**
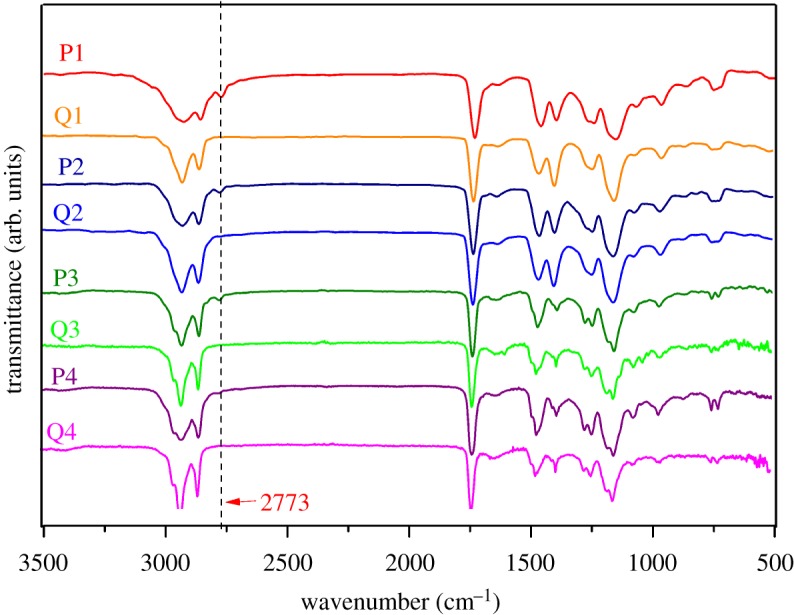
Comparison of infrared spectra for the starting neutral copolymers P1, P2, P3 and P4, and their corresponding quaternized forms Q1, Q2, Q3 and Q4.

Association of intermolecular chains, as well as the interactions, determine how elastomeric networks are formed [11,29]. In addition to the conventional construction of elastomeric networks where polymer chains are covalently cross-linked, supramolecular interactions, such as ionic interactions studied in this contribution, can act as dynamic cross-links and therefore give the resulting elastomers more processability via, for instance, solvoplastic property. The structures of as-prepared starting copolymers and their corresponding quaternized copolymers were investigated by both ^1^H NMR shown in the supporting information (electronic supplementary material, figures S1 and S2) and infrared spectroscopy shown in figure 2.

Because the tertiary amine in the starting polymer was quaternized, the positively charged nitrogen atom became more electrophilic and the steric stabilization of quaternary amine group became larger, both the strength and position of the bands of the methyl and methylene of amine were changed. All the peaks of the quaternized copolymers have obvious differences in strength from the starting copolymers; notably the band at 2773 cm^−1^, which belongs to the C–H stretching vibration of CH_2_ next to tertiary amino group, disappeared after quaternization, and the same change of the peaks has been found in other literature [30,31]. The results indicate the successful quaternization of PLMA-*co*-PDMAEMA, and essentially all the PDMAEMA components were quaternized.

### 3.2. Formation of gel network

**Scheme 2.**
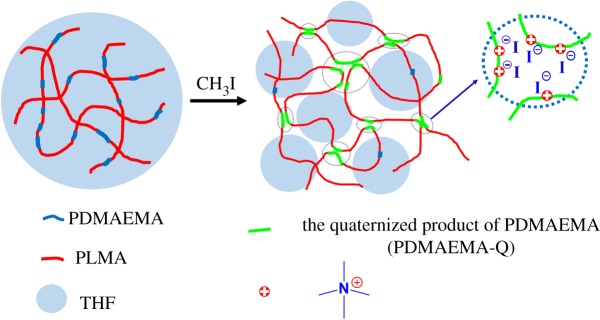
Illustration of the gelation of PDMAEMA-*co*-PLMA after selective ionization.

Three-dimensional polymer network is regarded necessary for the formation of gel, whether it is a hydrogel or organogel. Ionic polymers are often found to have strong association between the charged moieties driving the formation of a network [17,32]. In general, the driving [33] force of the formation of the gel can be divided into hydrogen bonds [34,35], halogen bonds [36], metal coordination [37], hydrophobic interaction [38] and π–π stacking or interaction [39]. In our study, after the addition of methyl iodide, quaternary ammonium groups are first formed. Since these units are insoluble in THF, and that the LMA segments remain soluble (table 2), a three-dimensional network was established through the ionic interaction between quaternized DMAEMA units, leading to the trapping of THF within this network and formation of a gel (scheme 2). The self-assembly of the ionic moieties of the quaternized copolymer does form a homogeneous molecular network where the LMA segments are interconnected by the DMq segments that act as ionic cross-links.

**Table 2.** Solubility of copolymer constituents in different solvents at 25°C.

polymer	THF	DMF	DMSO	hexane
PLMA	√	×	×	√
PDMAEMA	√	×	×	×
PDMAEMA-Q^a^	×	√	√	×
Q1	×	√	√	×
Q2	×	√	√	×
Q3	√	×	×	×
Q4	√	×	×	×

^a^Quaternized product of PDMAEMA. √, soluble; ×, insoluble or very slightly soluble.

### 3.3. Thermal properties

**Figure 3.**
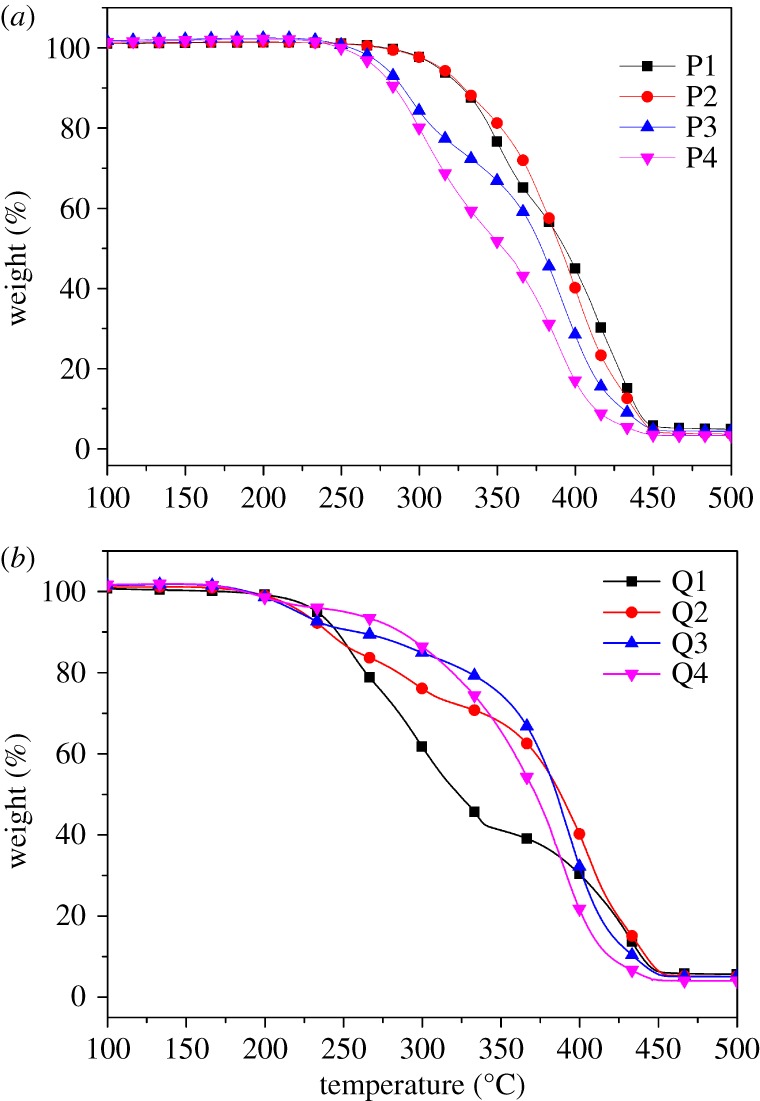
Thermogravimetric curves of copolymers under a nitrogen atmosphere with a heating rate of 10°C min^−1^. (*a*) Staring copolymers; (*b*) quaternized copolymers.

The decomposition temperature (*T*_d_) of the copolymers was defined as the temperature at 10% mass loss on the TGA curves [40]. For the starting copolymers (P1, P2, P3, P4), the *T*_d_ rapidly declines as the content of PDMAEMA decreases, as shown in figure 3. The decomposition temperature of P1, P2, P3 and P4 was at about 327°C, 322°C, 289°C and 284°C, respectively. In addition, all the *T*_d_'s of quaternized polymers were lower than those of the starting copolymers (245°C, 240°C, 259°C and 286°C for Q1–Q4). Therefore, all the polymers are stable in normal operating temperatures.

**Figure 4.**
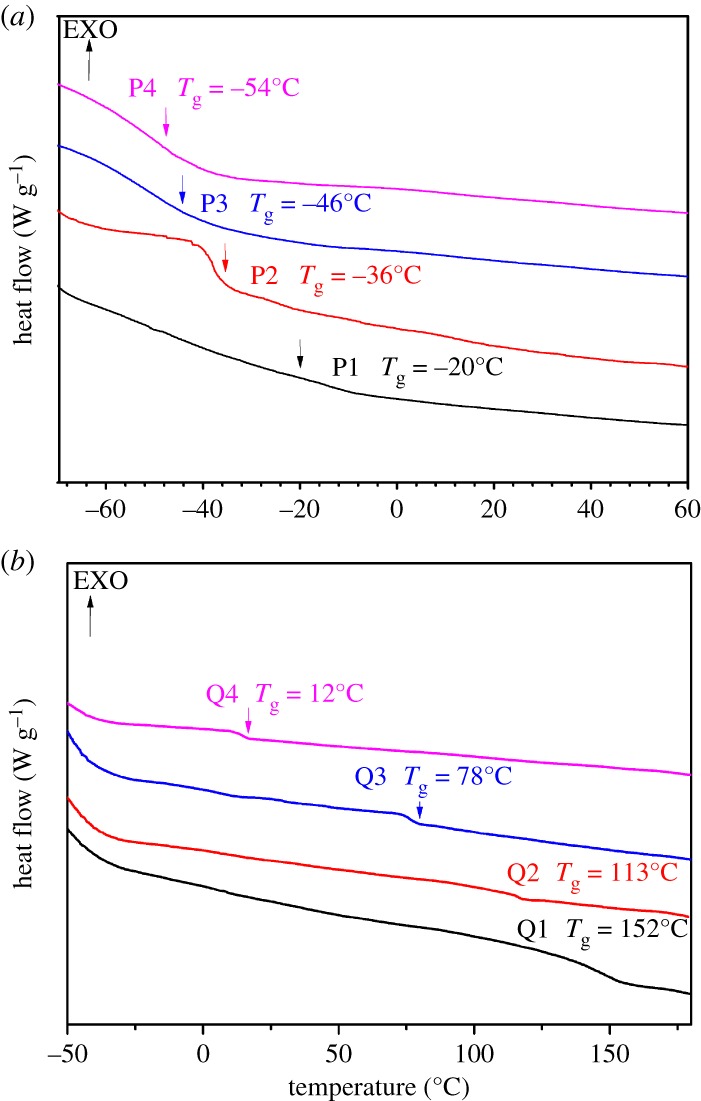
Heat flow curves from DSC measurements under a nitrogen atmosphere with a heating rate of 10°C min^−1^. (*a*) Staring copolymers; (*b*) quaternized copolymers.

The copolymers were synthesized by solution polymerization. The two methacrylate monomers have very similar reactivity, therefore a uniform distribution of the two components is expected. As for P1, whose molar ratio between PLMA and PDMAEMA was 1 : 1, a single glass transition was expected, as was seen in the DSC thermogram in figure 4. The *T*_g_ determined by the midpoint of the transition region for polymers P1 to P4 varied as a function of respective LMA content, from −20°C to −54°C. The observation of a single *T*_g_ for all the polymers further supports the uniform distribution of LMA and DMAEMA among the copolymer chain. At the same time, the compatibility of LMA and DMAEMA is also good. In addition, the *T*_g_ of the quaternized copolymers Q1–Q4 was 152°C, 113°C, 78°C, and 12°C respectively. It could be clearly seen that the *T*_g_ of quaternized copolymers was greater than starting copolymers since PDMAEMA formed ions. Furthermore, the difference of quaternized PDMAEMA content causes the gradual variation of the *T*_g_ [41]*.* Interactions of ions limited the movement of the molecular chain, leading to an increase in *T*_g_ of copolymers.

### 3.4. Rheological properties

According to preliminary tests, it is found that these ionic gels form at a fairly rapid rate. Because of this, all samples for rheological measurement were done within 3 min of the formation of the gel. Each concentration of quaternized copolymers for rheological properties is determined as the lowest gel-forming concentration of each sample. All the gels were formed within 180 s. The lowest gel-forming concentration is shown in electronic supplementary material, figure S5. Although there is no linear relationship between gel forming concentration and component ratio of PDMAEMA of copolymers, it was found that the concentration of each PDMAEMA-Q in THF was very similar (0.051 g ml^−1^, 0.049 g ml^−1^, 0.050 g ml^−1^ and 0.051 g ml^−1^ for Q1–Q4). The concentrations of copolymer for rheological properties and each component in polymer are shown in table 3.

**Table 3.** Concentration of copolymer component in the gel.

	concentration (g ml^−1^)		
sample	copolymer	LMA^a^	PDMAEMA-Q^a^
Q1	0.098	0.048	0.050
Q2	0.229	0.179	0.050
Q3	0.296	0.246	0.050
Q4	0.568	0.518	0.050

^a^As calculated by using the LMA weight per cent from ^1^H NMR results.

**Figure 5.**
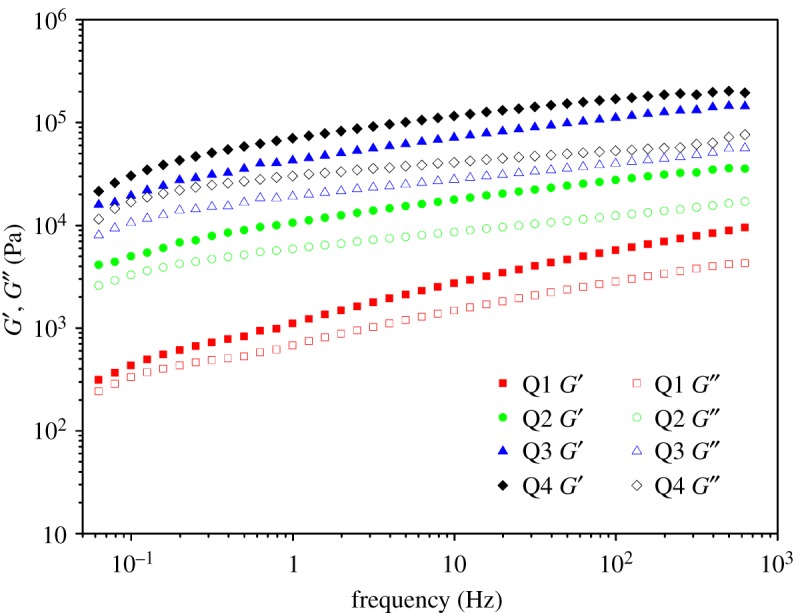
Storage modulus *G*′ and loss modulus *G^″^* as a function of angular frequency for the THF solutions of Q1 (0.098 g ml^−1^), Q2 (0.229 g ml^−1^), Q3 (0.296 g ml^−1^) and Q4 (0.568 g ml^−1^) at 0.1% strain at 25°C.

It is found that *G*′ is greater than *G*^″^ for all samples over the entire range of frequency in figure 5, which indicates that the elastic component of the dynamic modulus was greater, suggesting that the ion-gels act like a quasi-solid. The storage modulus *G*′ and loss modulus *G^″^* come from the contribution of two parts. One is the ions from the quaternized PDMAEMA, the other is LMA that has a relatively longer chain. A typical characteristic of physical ion-gel is the gel–sol phase transition, because the gel network is based on noncovalent interactions [42]. *G*′ is greater than *G*^″^ over the whole range explored, and the profiles of *G*′ and *G*^″^ are nearly parallel with only a slight frequency dependence. This shows that there was little change of internal structure of the ion-gel with changed frequency. This is somewhat different from other ion-gels where a structural change was observed [43]. In addition, it is found that *G*′ and *G*^″^ of Q2, Q3 and Q4 are obviously greater than those of Q1. This may be related to the concentration of the polymer. Although the elastic modulus will increase with polymer concentration, it is less significant at higher concentrations [44].

**Figure 6.**
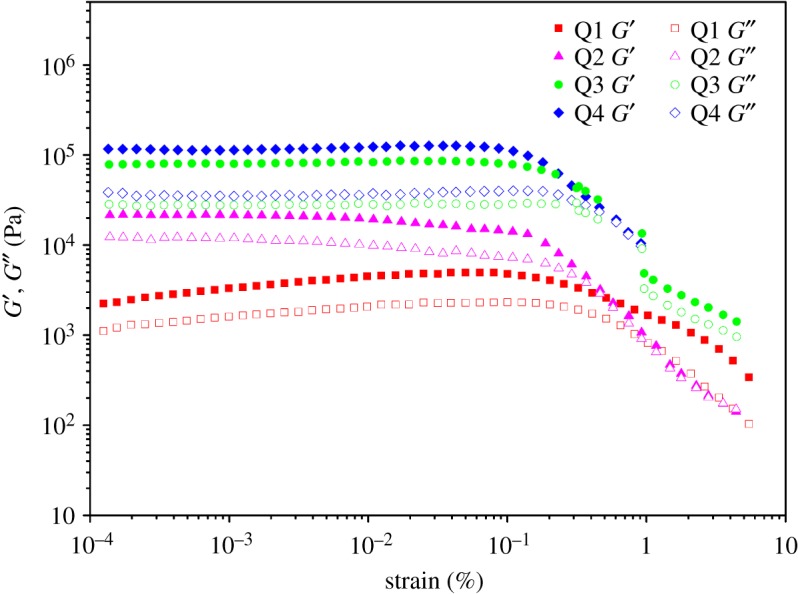
Storage modulus *G*′ and loss modulus *G^″^* as a function of strain for THF solutions of Q1, Q2, Q3 and Q4 at a frequency of 1 Hz at 25°C.

The strain amplitude sweeps also show the elastic response of the ion-gels, as shown in figure 6. Above 2% of deformation, the border of the linear viscoelastic regime is marked by a subsequent decrease in strongly nonlinear conditions. At this stage, the physically cross-linked network is subjected to some degree of damage, which is usually seen for such type of gels [45,46].

### 3.5. Mechanical properties

Mechanical properties are among the most important factors considered for practical applications of elastomers. The stress of Q4 gel in THF is relatively low, but a strong elastomer film was obtained after the evaporation of solvent. Elasticity was evaluated only for the quaternized random copolymer Q4, whereas Q1, Q2 and Q3 appeared to be too brittle and not elastomeric enough to allow extensive stretching, as shown in electronic supplementary material, figure S6. The brittleness of Q1, Q2 and Q3 films after solvent evaporation can be attributed to their high content of the quaternized PDMAEMA, leading to a high cross-link density and high *T*_g_. In fact, the percentage of quaternized PDMAEMA is so high that Q1 and Q2 have very poor solubility in THF, as shown in table 2. In addition, the unquaternized PDMAEMA has a *T*_g_ of 18°C [47], higher than that of PLMA (−70°C) [28], indicating the former is more rigid even if in its unionized state.

**Figure 7.**
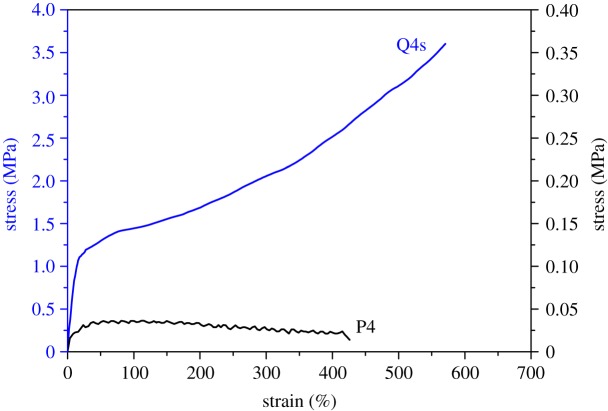
Stress–strain curves of P4 and Q4s in their bulk form measured at a stretching rate of 10 mm min^−1^.

The ionically cross-linked elastomer film Q4s was found to have an excellent elongation at break of over 600%, as shown in figure 7. The ultimate tensile strength of this film (nearly 3.8 MPa) is approximately 80 times higher as compared to the non-quaternized P4 film. Together with the fact that ionic interactions act as effective cross-links, it is likely that these supramolecular cross-links are in a dynamic state, where some cross-linking points are destroyed due to stretching while others form when at proper distance. The ionic cross-linking sites may interconnect with each other in a hop-on and hop-off manner that allows extra extension of polymer chains without over-sliding while maintaining the overall network structure, as evidenced by the nearly linear relationship from 50% to 600% on the stress–strain curve of figure 7, as well as the fact that the recovered samples are also stretchable. It is clear that the supramolecular interactions provide good strength for the material.

**Figure 8.**
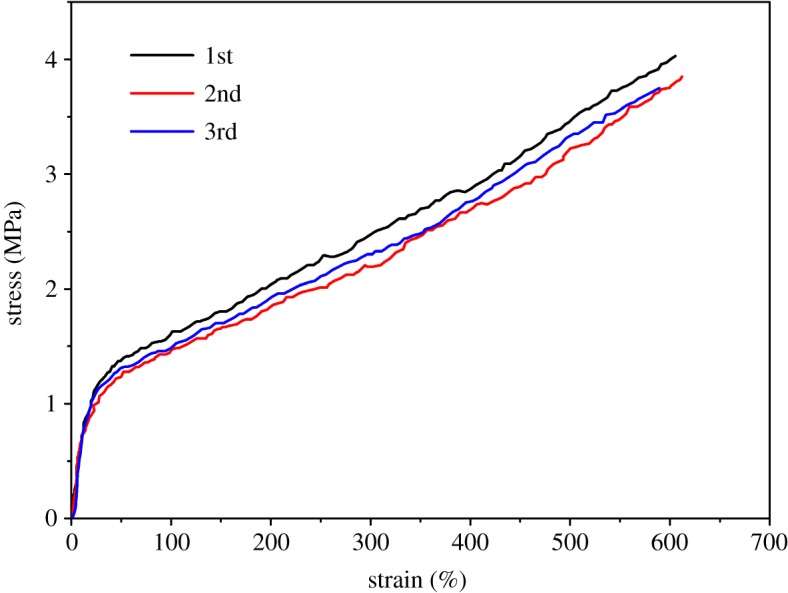
Stress–strain curves of Q4s in its bulk form measured at a stretching rate of 10 mm min^−1^. The legend 1st, 2nd and 3rd denotes the elastomer being dissolved–evaporated once, twice and three times.

To illustrate its recyclable character, the Q4s elastomer was dissolved and dried for several cycles. The three stress and strain curves of the redissolved and evaporated films are found to be very similar to each other as shown in figure 8. Particularly, both the elongation at break and stress at break are very similar between the samples. This confirms the recyclability of the elastomer, which is a major advantage as compared to conventional commercial chemically cross-linked elastomers, such as polydimethylsiloxane, that can only swell when exposed to organic solvents.

For a more quantitative comparison of elasticity of the samples, room temperature lost recovery measurements were made. The items are defined in equations (3.2) and (3.3):

3.2 $$\text{Strains}=\frac{\left(L-L_{0}\right)}{L}$$

and

3.3 $$\text{Lost}\;\text{recovery}=\frac{\left(L_{r}-L_{0}\right)}{\left(L-L_{0}\right)},$$

where *L*_0_ is the length of the film prior to stretching, *L* is the length to which the film was stretched, and *L*_r_ is the final length after relaxation. Each strain was tested only once per sample.

**Figure 9.**
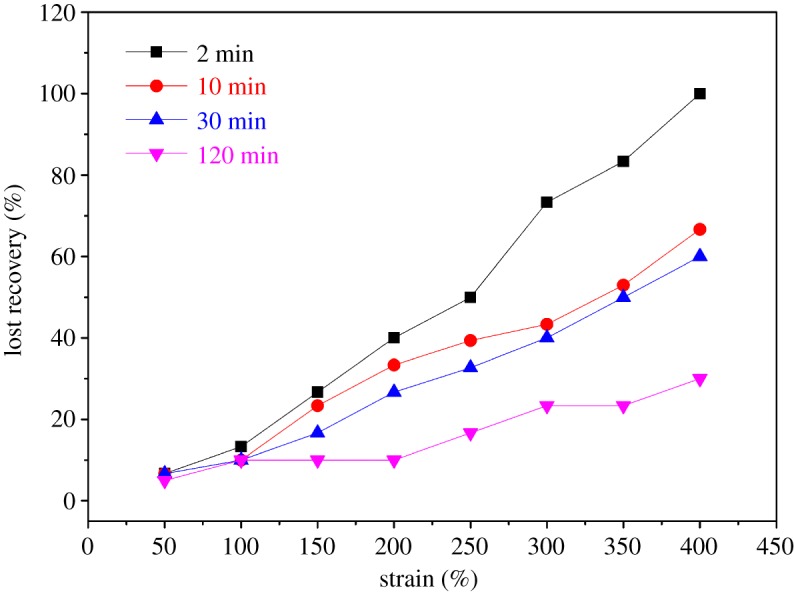
Lost recovery for films of Q4s stretched to various strains where they were held for 2 min and then allowed to relax with no external force for 2, 10, 30 and 120 min (all at 25°C).

The lost recoveries of Q4s are shown in figure 9. With the increase in recovery time, the per cent of recovery also increases, from 29.6% for 2 min to 101.3% for 120 min at the strain of 400%. Such tendency becomes less obvious at lower strain; for example, the lost recovery after relaxation of 120 min is 10.2% compared to 26.7% of 2 min at the strain of 150%. In the small strain range (below 100%), the lost recovery decreases with increasing relaxation time and reaches a minimum value of 13.2% at the longest waiting time in this study (≈120 min), suggesting a high elastic response of the material. On the other hand, there is a small plastic deformation at larger strains of about 350–400%. This is associated with the intrinsic character of supramolecular cross-linking. When the external force is applied, the movement of segments in the ion elastomer is affected by internal friction resistance. The movement of segment cannot keep up with the change of external force, so that the strain is lagging behind the stress where the mechanical energy was transformed into heat. As a result, the specimen cannot fully retract after removing the external force. In addition, because of the physical character of the cross-linking for the elastomer, connecting points are constantly being destroyed and rebuilt in the process of stretching, much like in the event of molecular chain slip, where new physical cross-linking points are also re-established. Since the strength of the physical cross-linking point is lower than that of the chemical cross-linking, the deformation recovery is somewhat compromised.

## 4. Conclusion

We have designed and synthesized a series of ion-gels and their corresponding recyclable solvoplastic elastomers when in bulk form with physically supramolecular cross-linking through ionic interactions. The starting random copolymers were synthesized from LMA, whose homopolymer has a low *T*_g_, and PDMAEMA, which could be quaternized. The formation of these ion-gels is fast and efficient, with a typical gelation time of 180 s. Rheological properties were found to be highly dependent on the content of LMA. With the increase in LMA, storage modulus *G*′ and loss modulus *G^″^* of the copolymer gels increase. After the evaporation of solvent of the gel, physically cross-linked elastomer with good elasticity was obtained for which the mass ratio of LMA and DMAEMA-Q is 10 to 1, and the ultimate tensile strength (3.8 MPa) is 80 times higher as compared to corresponding non-quaternized film, although a certain permanent chain relaxation was also observed after repeated tensile test cycles possibly due to the dynamic character of the ionic cross-links. In addition to good elastic modulus, the elastomer is also solvoplastic and recyclable as evidenced by dissolution in certain solvents such as DMF. The recyclable characters of the ion-gels and elastomeric ionomer when in bulk form have large potentials for their applications in wearable devices and as composite materials increasing the elasticity of other materials. Functionality, such as potential conductivity, and mechanical properties can also be adjusted by varying the co-monomer ratio, which is readily implementable.

## Supplementary Material

Supplementary monomer feed ratio, 1H NMR, photos of the quaternized copolymers.

## Data accessibility

Data are available from the Dryad Digital Repository: http://dx.doi.org/10.5061/dryad.d40s45p [48].
