# Supplementary material for: Fast formation of a supramolecular ion gel/solvoplastic elastomer with excellent stretchability
Source: R Soc Open Sci. 2018 Jun 20;5(6):180271. doi: 10.1098/rsos.180271 (PMC6030259; doi:10.1098/rsos.180271)
Supplement: Supplementary monomer feed ratio, 1H NMR, photos of the quaternized copolymers. [file rsos180271supp1.docx]

*Supplementary information for:*

Fast Formation of a Supramolecular Ion Gel/ Solvoplastic Elastomer with Excellent Stretchability

*Shishun Bai*^1^*, Xin Wang*^1^**, Jaana Vapaavuori*^2^*, Xianru He*^1^***

1. School of Materials Science and Engineering, Energy Polymer Research Center, Southwest Petroleum University, 8 Xindu Avenue, Chengdu, Sichuan 610500, China.

2. Département de chimie, Université de Montréal, C.P. 6128, succursale Centre-Ville, Montréal, QC, Canada H3C 3J7

*Corresponding author. Email: [xin.wang@swpu.edu.cn](mailto:xin.wang@swpu.edu.cn) (X.W.), [xrhe@swpu.edu.cn](mailto:xrhe@swpu.edu.cn%20)  (X.H.)

**Table S1.** **Composition and molecular weights of the starting P(DMAEMA-*co*-LMA) copolymers**

| polymer | wt % DMAEMA content | | *M_n_*_，GPC_(× 10^4^) | *M*_w_, _GPC_ (× 10^4^) | PDI |
| --- | --- | --- | --- | --- | --- |
|  | theoretical | ^1^H NMR |  |  |  |
| P1 | 50.0 | 51.0 | 19.35 | 43.09 | 2.22 |
| P2 | 25.0 | 24.3 | 17.85 | 45.46 | 2.54 |
| P3 | 16.7 | 16.8 | 15.39 | 33.54 | 2.18 |
| P4 | 9.1 | 8.8 | 15.37 | 45.99 | 2.99 |

**Figure S1.** ^1^H NMR spectrum of P(DMAEMA-co-LMA) copolymer P1 in CDCl_3_.

**Figure S2.** Comparison of ^1^H NMR spectrum of copolymer P1, P2, P3 and P4 in CDCl_3_.

**Figure S3.** ^1^H NMR spectrum of the quaternized copolymer Q1 in DMF-*d_7_*.

**Figure S4.** Comparison of ^1^H NMR spectrum of quaternized copolymer Q1, Q2, Q3 and Q4 in DMF-*d_7_*.

**Table S2.** **Degree of quaternization of the starting copolymers**

| polymer | degree |
| --- | --- |
| Q1 | 92% |
| Q2 | 97% |
| Q3 | 95% |
| Q4 | 96% |


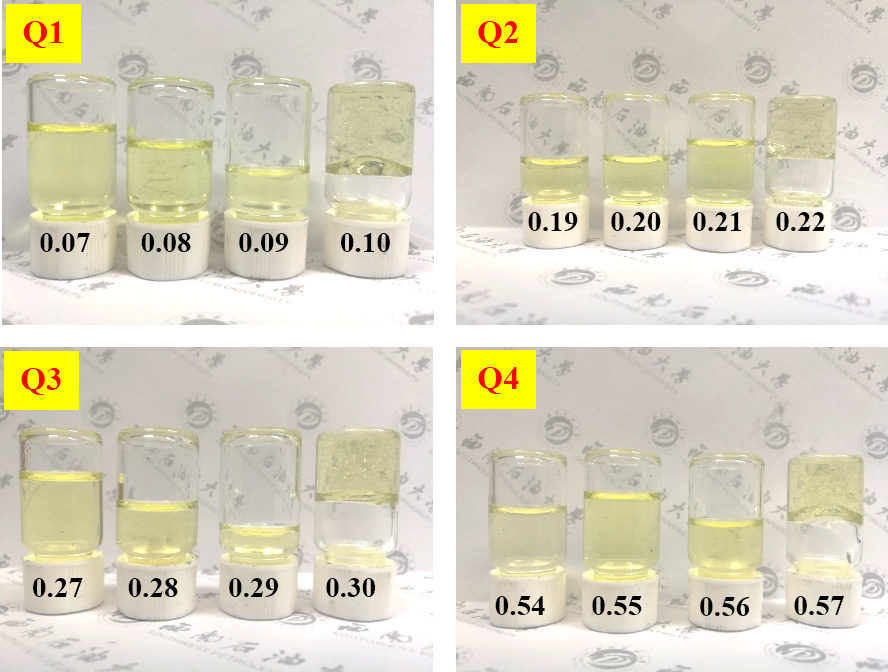


**Figure S5.** Photos of the quaternized copolymers Q1, Q2, Q3 and Q4 with different concentration (g/mL) in THF at 25 ^o^C.

**
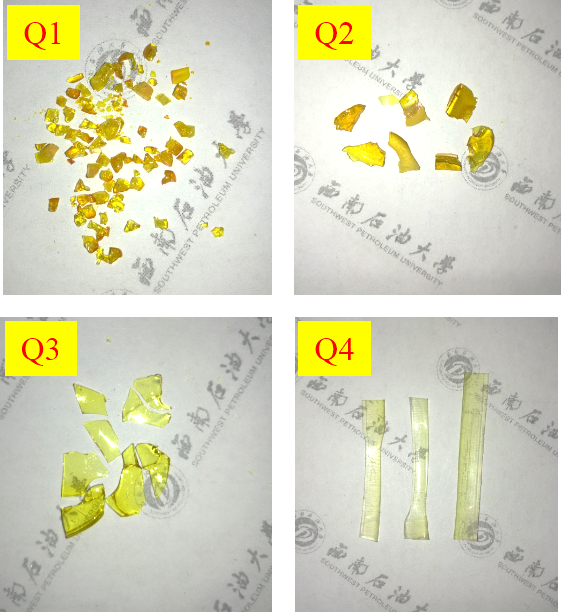
**

**Figure S6.** Photos of the quaternized copolymers Q1, Q2, Q3 and Q4 after solvent evaporation.
